# miR‐322‐5p targets IGF‐1 and is suppressed in the heart of rats with pulmonary hypertension

**DOI:** 10.1002/2211-5463.12369

**Published:** 2018-01-24

**Authors:** Martin Connolly, Benjamin E. Garfield, Alexi Crosby, Nick W. Morrell, Stephen J. Wort, Paul R. Kemp

**Affiliations:** ^1^ Molecular Medicine National Heart & Lung Institute Imperial College London UK; ^2^ National Institute for Health Research Respiratory Biomedical Research Unit at Royal Brompton and Harefield NHS Foundation Trust and Imperial College London UK; ^3^ Department of Medicine Addenbrookes Hospital University of Cambridge UK

**Keywords:** IGF‐1, miR‐322/424, pulmonary arterial hypertension, right ventricular hypertrophy

## Abstract

Pulmonary arterial hypertension (PAH) is characterised by remodelling of the pulmonary vasculature leading to right ventricular hypertrophy. Here, we show that miR‐322‐5p (the rodent orthologue of miR‐424‐5p) expression is decreased in the right ventricle of monocrotaline‐treated rats, a model of PAH, whereas a putative target insulin‐like growth factor 1 (IGF‐1) is increased. IGF‐1 mRNA was enriched 16‐fold in RNA immunoprecipitated with Ago2, indicating binding to miR‐322‐5p. In cell transfection experiments, miR‐322‐5p suppressed the activity of a luciferase reporter containing a section of the IGF‐1 3′ untranslated region (UTR) as well as IGF‐1 mRNA and protein levels. Taken together, these data suggest that miR‐322 targets IGF‐1, a process downregulated in PAH‐related RV hypertrophy.

**Abbreviations**
AAV: adeno‐associated virus
Ago2: argonaute 2
Akt: protein kinase B
Bcl‐2: B‐cell lymphoma 2
Cdc: cell division control protein
ELISA: enzyme‐linked immunosorbent assay
FBS: fetal bovine serum
IGF‐1: insulin‐like growth factor‐1
IGF‐1R: insulin‐like growth factor‐1 receptor
LAD: left anterior descending
MCT: monocrotaline
miRNA: microRNA
mRNA: messenger RNA
mTOR: mechanistic target of rapamycin
PAH: pulmonary arterial hypertension
PBS: phosphate‐buffered saline
PI3K: phosphatidylinositide 3‐kinase
PPAR‐γ: peroxisome proliferator‐activated receptor‐γ
RhoA: Ras homolog *gene* family, member A
RISC: RNA‐induced silencing complex
RV: right ventricle
s.c.: subcutaneous
UTR: untranslated region

Pulmonary arterial hypertension (PAH) is a pathophysiological process characterised by high, precapillary pulmonary vascular resistance 1. PAH can be idiopathic, heritable (mainly associated with defects in bone morphogenetic protein receptor 2) or associated with an underlying condition 2. The pathology of PAH is largely consistent irrespective of aetiology and involves significant vascular remodelling and restriction causing a reduction in cross‐sectional area to pulmonary blood flow, which can lead to right ventricular hypertrophy 3 and failure 4. Prognosis for patients varies, with average survival rates of 58–67% 3 years after diagnosis for idiopathic PAH 5, 6, with cause of death normally being attributed to right ventricular failure 7. One widely used model of PAH is monocrotaline (MCT) treatment of rats 8. In this model, following a single dose of MCT the animals develop elevated pulmonary vascular resistance leading to right heart hypertrophy within 4 weeks 9.

Under normal conditions, adult heart size is maintained but can change in response to either increased or reduced load resulting in hypertrophy or atrophy, respectively. As adult cardiac cells do not divide, change in heart size is achieved by altering the size of cardiac myocytes rather than their number. The relative rates of protein synthesis and breakdown contribute to the maintenance of cell size 10, 11. When these are in balance, cell size is maintained; consequently, the RV cardiomyocyte hypertrophy observed in PAH requires the rate of protein synthesis to exceed that of protein breakdown.

Insulin‐like growth factor‐1 is a growth factor that promotes synthesis and inhibits protein breakdown in both cardiac and skeletal muscle. Following binding to its receptor, IGF‐1 activates protein kinase B (Akt) via phosphatidylinositide 3‐kinase (PI3K) stimulation 12. This signalling system contributes to physiological cardiac hypertrophy, with knockout animals for both Akt and PI3K showing attenuated heart enlargement 13, 14. Furthermore, mice that overexpress a dominant active PI3K demonstrated increased heart size that did not become maladaptive 15. However, it is possible that IGF‐1 also contributes to pathological hypertrophy in combination with other factors, as treatment of salt‐sensitive Dahl rats with IGF‐1 reduced survival and promoted left ventricular dysfunction 16. Furthermore, IGF‐1 deficiency alleviates cardiac hypertrophy in a model of abdominal aortic constriction 17.

The signalling pathways which contribute to protein turnover and cell maintenance are regulated in part by microRNAs, short noncoding RNAs that downregulate protein expression via translational repression or mRNA degradation 18. The roles of many miRNAs in the regulation of cardiac mass have previously been studied; for instance, knockdown of miR‐133, which targets Cdc42 and RhoA, causes significant hypertrophy *in vivo* 19, whereas inhibition of miR‐27b, which targets peroxisome proliferator‐activated receptor‐γ (PPAR‐γ), attenuates cardiac hypertrophy 20. miR‐322‐5p is the rodent orthologue of miR‐424‐5p, a miRNA from the 424/542 cluster located on the X chromosome, which has been shown to target the insulin‐like growth factor 1 receptor (IGF‐1R) in mammary tissue 21 and cardiac muscle 22. Expression of miR‐322 has previously been shown to be reduced in the lung of monocrotaline‐treated rats. Following intranasal delivery of miR‐322 to the rats, RV systolic pressure and RV weight were shown to be significantly reduced compared to controls, suggesting restoring expression could ameliorate the effects of PAH on the RV 23. Importantly, expression of the miRNA in the hearts of these animals was not reported.

Insulin‐like growth factor‐1 is a predicted target for miR‐322‐5p based on database algorithms, which match miRNA sequences to putative mRNA targets. We therefore hypothesised that MCT treatment would alter miR‐322 expression in the right ventricle and contribute to hypertrophy by increasing IGF‐1 expression.

## Materials and methods

### Monocrotaline rat model

Monocrotaline or PBS was administered to 6‐ to 8‐week‐old rats via s.c. injection (40 mg·kg^−1^), and 4 weeks later, pulmonary arterial pressure was measured as previously described before the animals were humanely sacrificed 24. The hearts were removed, the right ventricle was dissected free from the left ventricle and septum and both components were weighed and then snap‐frozen.

### RNA extraction and RT‐qPCR

Right ventricles were crushed in TRIzol for RNA extraction as previously described 25. Cell RNA extractions were performed using the TaKaRa CellAmp Direct RNA kit (Clontech Saint‐Germain‐en‐Laye, France) as per the manufacturer's protocol. For miR‐322‐5p measurements, RNA was reverse‐transcribed (RT) using miR‐322‐5p and U6 Applied Biosciences TaqMan primers suspended in 1x TE buffer as per the manufacturer's instructions. cDNA was used for qPCR with TaqMan fluorescent probes for miR‐322‐5p and U6 according to the manufacturer's instructions. mRNA was measured via Omniscript RT (Qiagen, Manchester, UK) with random primers (Promega, Madison, WI, USA) as per the manufacturer's protocol. cDNA was diluted 1/10 with deionised water and used in qPCRs with QuantiFast SYBR Green (Qiagen) and specific primers (Sigma, Poole, Dorset, UK) for targets of interest as previously described 26.

### Cell culture and transfection

C2C12 mouse myoblasts were cultured in DMEM (Sigma) supplemented with 10% fetal bovine serum (FBS) and 1% Pen‐Strep (Gibco, Gaithersburg, MD, USA) as previously described 27; LHCN‐M2 human myoblasts were cultured in human skeletal muscle medium (PromoCell, Heidelberg, Germany) supplemented with 20% serum (15% FBS and 5% manufacturer's serum) as previously described 28. Cells were transfected once ~70% confluent with miRNA mimic and/or antagomir (miRVana) and Lipofectamine 2000 (Invitrogen, Paisley, UK) using Opti‐MEM (Gibco) as recommended by the manufacturer and as previously described 27. Transfected miR‐mimic concentrations were kept the same in all reactions by addition of control miRNA mimic as appropriate.

### Protein isolation from cells and RISC immunoprecipitation

Forty‐eight hours after transfection, C2C12s were lysed in 1x cell lysis buffer (Cell Signalling Technologies, Hitchin, UK) supplemented at 1/100 with protease inhibitor cocktail (Sigma). Samples were centrifuged at 12 000 ***g*** and supernatant transferred to clean microcentrifuge tubes. Protein‐G‐bound Sepharose beads were added to the lysate, and the tubes were rotated for 2 h at 4 °C for lysate preclearing. Beads to be used for the pull‐down were blocked using 1 mg·mL^−1^ salmon sperm DNA (Sigma) under rotation for 2 h at 4 °C. Blocked beads were washed in cell lysis buffer and the precleared lysate added. Antibodies for the immunoprecipitation, anti‐Ago2 (Millipore, Watford, UK) or control anti‐mouse IgG (Invitrogen), were added at matching concentrations of 1 : 100 and the samples rotated overnight at 4 °C. Beads were then washed several times in cell lysis buffer and PBS before TRIzol RNA extraction as detailed above.

### Secreted IGF‐1 measurements (western blot and ELISA)

Forty‐eight hours after transfection, cells were placed into serum‐free medium for 24 h. The medium was collected and cleared of cell debris by centrifugation at 12 000 ***g*** at 4 °C for 20 minutes. The supernatant was transferred to new tubes and kept on ice. Secreted IGF‐1 quantification was performed first by ELISA (R&D Systems, Abingdon, UK) as per the manufacturer's instructions and secondly via western blot following acetone precipitation (as previously described 25). Total protein concentration in the collected medium was determined via Bradford assay (Bio‐Rad, Watford, UK). ELISA IGF‐1 values were then normalised to the total secreted protein, and western blot images were normalised to Ponceau S‐stained membranes.

### pMIR‐REPORT luciferase assay

A section of the 3′UTR of IGF‐1 was TA‐cloned into pGEM‐T Easy (Promega) using primers: forward (5′–3′) GGGACTAGTGAGGAGCCTCCCACGGAGCA, reverse: (5′–3′) CCCACTAGTGCTACGTGGGAAGAGGTGAAG. SpeI digestion was performed and the UTR sequence ligated into a pMIR‐REPORT expression vector (ThermoFisher). Final products were confirmed by sequencing. For luciferase assays, cells were transfected with luciferase reporter vectors (pRLTK used as a control) 24 h after miRNA transfection using standard Lipofectamine (Invitrogen) and the assay performed as previously described 27.

### Statistics

Animal experiments were conducted in two groups for a total of nine MCT‐treated and nine PBS‐treated animals. Differences between groups were calculated using Student's *t*‐test for normally distributed data or by Mann–Whitney *U*‐test for nonparametric data (GraphPad PRISM, La Jolla, CA, USA). *In vitro* mRNA expression data shown were produced in three independent experiments, with each experiment consisting of six independent transfections assayed in duplicate. Box plots are expressed as median with min–max bars. *In vitro* protein expression data shown via western blot are three independent experiments from six‐well plates, and via ELISA are three independent experiments, with each with each experiment consisting of six independent transfections. RISC IP was performed twice.

## Results

### MCT rat model: miR‐322 and IGF‐1 are inversely expressed in the RV

**Figure 1:**
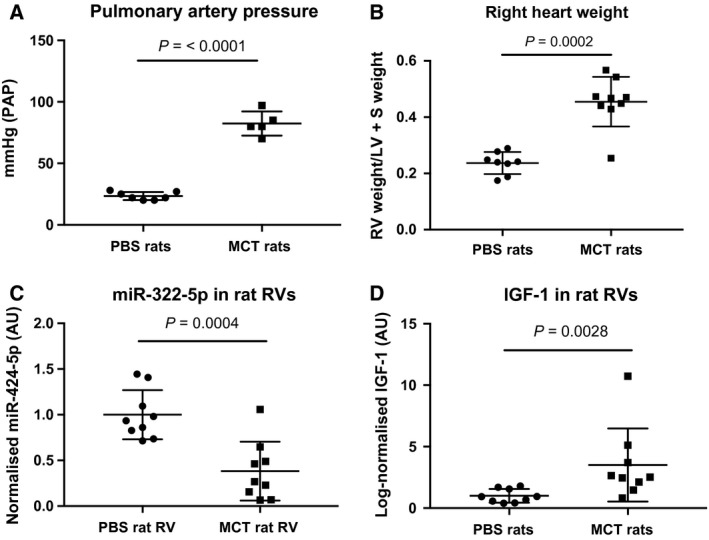
(A) Pulmonary arterial pressure measured as mm of mercury (Hg) in five monocrotaline (MCT)‐treated and seven PBS‐treated rats. (B) Right ventricle weight normalised to left ventricle and septum weight in nine MCT‐treated and eight PBS‐treated rats. (C) Normalised miR‐322‐5p expression in MCT‐ and PBS‐treated rats. D. IGF‐1 expression levels in MCT‐ and PBS‐treated rats.

Nine MCT‐treated and nine PBS‐treated rats were sacrificed after 4 weeks and markers of disease severity measured to determine PAH development in the two groups. Pulmonary arterial pressure (PAP) was higher in the MCT‐treated animals compared to controls suggesting increased vascular resistance (Fig. 1A). Right heart weight (right ventricle/(left ventricle + septum)) was higher in the MCT rats compared to controls, confirming the development of RV hypertrophy (Fig. 1B). Quantification of miR‐322‐5p in the right ventricles (RVs) showed a 2.6‐fold decrease (*P* = 0.0004) in miRNA expression in MCT‐treated rats compared to PBS‐treated controls (Fig. 1C). Conversely, IGF‐1 expression was significantly increased in the RVs of MCT rats compared to PBS controls (3.5‐fold, *P* = 0.0028), suggesting an upregulation of IGF‐1 signalling promoting growth (Fig. 1D).

### miR‐322 directly targets IGF‐1

**Figure 2:**
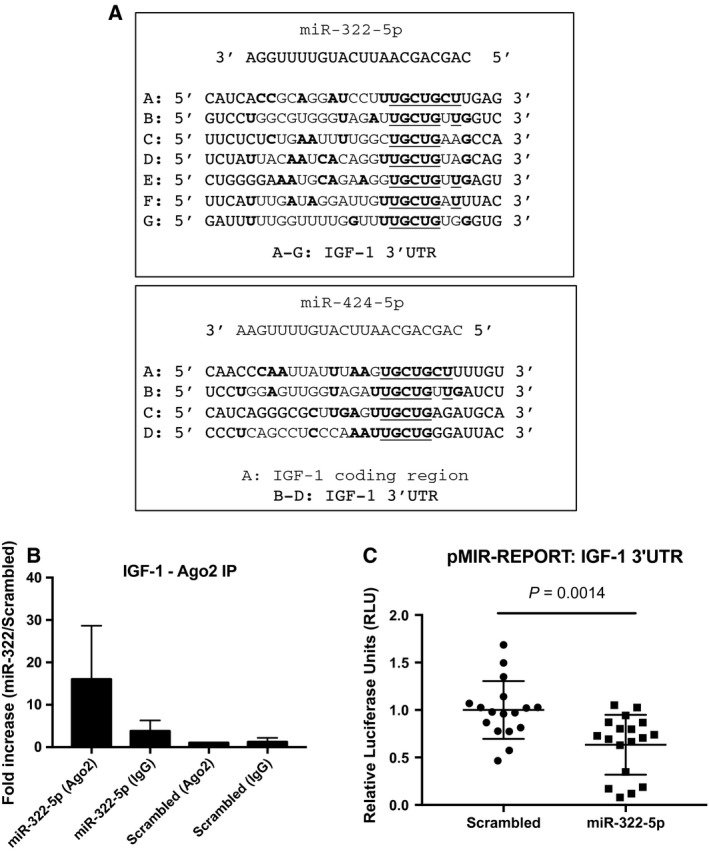
(A) (top) miR‐322‐5p mature sequence showing putative bind sites to IGF‐1 3′UTR with complimentary bases bolded and seed sequence match site underlined; (bottom) miR‐424‐5p mature sequence showing multiple bind sites to IGF‐1 coding region and 3′UTR with complimentary bases bolded and seed sequence match site underlined. (B) qPCR‐measured enrichment of mRNA from immunoprecipitation of argonaute 2 (Ago2) protein in cells transfected with miR‐322‐5p compared to control miRNA‐transfected cells. (C) Expression of pMIR‐REPORT luciferase with IGF‐1 3′UTR sequence in cells transfected with miR‐322‐5p compared to scrambled controls.

Bioinformatic analysis predicted IGF‐1 to be a target of both miR‐424‐5p and its mouse orthologue miR‐322‐5p. The mouse IGF‐1 gene has seven potential binding sites for miR‐322‐5p in its 3′UTR, shown in Fig. 2A. Region A was used for reporter cloning as the entire seed sequence is complimentary in this section. The human IGF‐1 gene has three potential binding sites for miR‐424‐5p in the UTR, and one in the coding region, a feature more commonly found in miRNA containing a 5′ AGCAGC motif 29 (Fig. 2A). To determine whether miR‐322‐5p targets IGF‐1 in myoblasts, we performed an Ago2 immunoprecipitation followed by RT‐qPCR using cells transfected with a miR‐322‐5p mimic or a scrambled control. This analysis showed IGF‐1 mRNA was enriched >16‐fold in RNA immunoprecipitated from miR‐322‐5p‐transfected cells compared to controls (Fig. 2B). To confirm targeting of IGF‐1, the region of the 3′UTR of IGF‐1 containing the putative miRNA‐binding site was cloned into pMIR‐REPORT (Fig. 2A) and the effect of miR‐322‐5p on luciferase activity determined in C2C12 cells. Luciferase activity was significantly reduced in miR‐322‐5p‐transfected cells compared to controls (Fig. 2C), confirming miR‐322‐5p binding to the 3′UTR of IGF‐1.

### miR‐322‐5p reduces IGF‐1 mRNA expression

**Figure 3:**
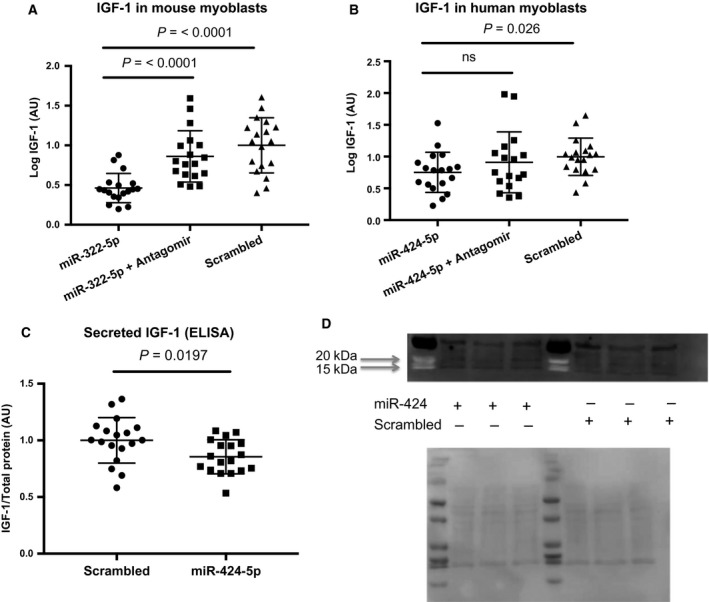
(A) IGF‐1 expression in C2C12 mouse myoblasts treated with miR‐322‐5p, miR‐322‐5p + antagomir inhibitor, or control miRNA. (B) IGF‐1 expression in LHCN‐M2 human myoblasts treated with miR‐424‐5p, miR‐424‐5p + antagomir inhibitor, or control miRNA. (C) Secreted IGF‐1 protein levels normalised to total secreted protein in LHCN‐M2 human myoblasts transfected with miR‐424‐5p or control miRNA. (D) Western blot showing protein levels of IGF‐1 in cells transfected with miR‐424‐5p (left) and control miRNA (right). Ponceau S staining of membrane shown below.

To determine the effect of miR‐322‐5p on IGF‐1 expression, we transfected mouse C2C12 cells as described in Methods. qPCR showed a significant reduction in IGF‐1 mRNA in miR‐322‐5p‐mimic‐transfected cells, and this effect was reversed by cotransfection with an antagomir (Fig. 3A). To determine whether human IGF‐1 was also targeted by the miRNA, LHCN‐M2 cells were transfected with miR‐424‐5p mimic. Again the expression of IGF‐1 was suppressed by miR‐424‐5p, but this suppression was not as large as that obtained in murine cells. Although median expression was higher in the presence of the antagomir than in the miR‐mimic‐only‐transfected cells, this difference did not reach statistical significance (Fig. 3B).

### miR‐424‐5p reduces IGF‐1 protein expression

To determine the effect of the miRNA on secreted IGF‐1 protein levels, we transfected human myoblasts with miR‐424‐5p. Forty‐eight hours later, growth medium was replaced with serum‐free medium for 24 h. The medium was aspirated and an ELISA performed for human IGF‐1. Consistent with reduced mRNA, IGF‐1 protein levels were reduced in miR‐424‐5p‐transfected cells compared to scrambled controls (Fig. 3C). A similar reduction in secreted IGF‐1 was observed by western blotting of separate samples (Fig. 3D).

## Discussion

Our data show that the hypertrophied RV from MCT‐treated rats had significantly reduced levels of miR‐322‐5p and elevated expression of IGF‐1. Furthermore, we show that miR‐322‐5p binds to the 3′UTR of IGF‐1 and suppresses IGF‐1 mRNA expression leading to a reduction in both IGF‐1 mRNA and protein. Together, these data imply that suppressed miR‐322‐5p in the heart of MCT rats may contribute to hypertrophy in part by increasing the expression of IGF‐1.

Insulin‐like growth factor‐1 signalling is well established as an important promoter of protein synthesis and a repressor of protein degradation. Through the IGF‐1 receptor, IGF‐1 promotes the activation of Akt and mTOR, both of which are master regulators of protein synthesis (reviewed in 30). Indeed, it has been shown that treatment of isolated cardiac cells with 50–100 nm IGF‐1 causes a 70% increase in protein synthesis over insulin treatment 31. The myocardium produces IGF‐1 locally, and evidence suggests that its activity not only promotes growth, but also has a protective effect against myocardial apoptosis as shown in a model of myocardial ischaemia and reperfusion 32. Thus, IGF‐1 functions to prevent myocyte loss and to increase the size of cells 33, contributing to cardiac hypertrophy both in normal cardiac growth and in disease states.

The reduction in RV miR‐322‐5p expression we observed differs from previously reported changes in miR‐322‐5p in cardiac hypertrophy. For example, following 4‐week aortic banding, miR‐322 was found to be elevated in the hypertrophying left ventricles of mice; however, this same study saw no significant increase in miR‐424‐5p in humans with end‐stage hypertrophic cardiomyopathy 34. Another study, in which rats had left anterior descending (LAD) artery ligations causing development of an ischaemic zone 35, noted elevated miR‐322‐5p expression in cardiac tissue compared to sham‐operated controls 36. Other studies on PAH have also measured miR‐322‐5p. For example, in response to MCT, Gubrij *et al*. 37 showed a reduction in miR‐322‐5p in the lung 3 weeks after treatment as well as a small but significant reduction in the RV. These effects were similar to those of Schlosser *et al*. 38 who showed significantly reduced lung miR‐322‐5p and lower mean RV miR‐322‐5p, although this did not reach statistical significance. The reductions observed were smaller than that which we found, but both the time point and the dose of MCT used in those studies vary from the ones used here. Unlike MCT treatment, hypoxia‐induced PAH appears to increase RV miR‐322‐5p in mice and to not change it in rats, suggesting that the changes in miRNA profile are model‐specific 38. The actual changes in the expression of this miRNA in the RV in humans with PH remain to be determined. The increase in miR‐322‐5p observed in the hypoxia models is consistent with the observations that miR‐322/424 is a hypoxia‐induced miRNA, which has been shown to be upregulated in cardiomyocytes during hypoxia‐induced apoptosis, and knockdown of the miRNA had a cytoprotective effect 39.

Taken together, the variable expression of miR‐322‐5p/miR‐424‐5p in hypertrophy suggests that it is under a complex control with a number of both positive and negative inputs and the measured expression level in the heart will be a consequence of all of these inputs. In our model, it is possible that the reduction in miR‐322‐5p is a consequence of MCT treatment, directly or as a consequence of increased afterload, and that this reduction contributes to the RV hypertrophy in part by elevating IGF‐1. However, as we picked a single time point for our study (4 weeks after treatment), we cannot determine whether miR‐322‐5p expression was suppressed prior to the increase in IGF‐1 or whether there was a transient increase in miR‐322‐5p. It is therefore possible that IGF‐1 itself suppresses the expression of miR‐322‐5p to reduce the pro‐apoptotic effects of this miRNA on cardiomyocytes 39. Such an effect would cause a positive feedback as IGF‐1 suppressed miR‐322‐5p, which then allowed greater translation of IGF‐1 sustaining elevated IGF‐1.

Such an explanation gains some support from a recent study investigating circulating levels of miR‐322‐5p in response to MCT 40. This study showed that miR‐322‐5p in circulation is elevated 1 week after treatment and then decreases at 2 weeks only to increase again by 6 weeks. However, the source of the miR‐322‐5p in circulation is not clear as miR‐424 expression is not restricted to the heart with expression also found in the skeletal muscle, a more abundant tissue than cardiac muscle.

Previous studies in the heart and other tissues have shown that miR‐322 contributes to the regulation of IGF‐1 signalling. For example, miR‐424 was shown to regulate the expression of the IGF‐1 receptor (IGF‐1R) in epithelial cells as part of the regulation of mammary involution 21. The targeting of the IGF‐1R was confirmed in a study in which miR‐322 or a miR‐322 sponge was expressed using an adeno‐associated virus (AAV) in the heart of diabetic or high‐fat‐diet‐fed mice. In this study, overexpression of the miRNA led to a reduction in IGF‐1R and sirtuin 4, key components of the insulin signalling pathway, as well as a reduction in Akt phosphorylation 22. Our data add to this study by indicating that IGF‐1 is an additional target of the miRNA, suggesting that reduced expression of this miRNA will help to promote cardiac hypertrophy by increasing the level of both IGF‐1 and its receptor. It remains to be seen which of these facets of the biology of IGF‐1 has the larger effect on cardiac growth.

Changes in miR‐322‐5p are not the only ones that occur in cardiac hypertrophy that are likely to affect the IGF‐1 signalling system. For example, miR‐223 is a miRNA that is suppressed by hypoxia but targets the IGF‐1R 41. Overexpression of this miRNA attenuated cardiac hypertrophy and suppressed IGF‐1R expression in analogous way to the effects of miR‐322‐5p in response to MCT. However, it is interesting to note that this miRNA is regulated in the opposite direction to miR‐322‐5p in response to hypoxia but targets at least one of the same genes, highlighting the complexity of the system and the fact that multiple miRNAs contribute to the regulation in individual proteins 41. Such complexity may account for the differences in the observed changes in miR‐424‐5p expression in PAH models.

The data also raise the potential of increasing miR‐424‐5p expression in the heart to reduce cardiac hypertrophy at least in PAH. Ideally, this would be achieved by a selective upregulation of the miRNA in the heart. Increasing miRNA is more difficult than suppressing their activity due to the processes involved in incorporating the miRNA into the RISC complex. However, mimics are currently being used in clinical trials for a range of conditions including cancer (reviewed in 42). Furthermore, direct cardiac injection of miRNA mimics for miR‐199a‐3p and miR‐590‐3p has been shown to enable myocardial repair 43 An alternative to mimics is the use of viral delivery of an expression cassette for the miRNA as described above for miR‐322‐5p. However, given the potential for dynamic changes in miR‐424 described previously, the development of this as an approach to reduce hypertrophy in PAH would require a much more detailed analysis of miR‐424‐5p expression in the heart during the development of disease. Finally, any potential reduction in RV hypertrophy has to be balanced against reduction in pulmonary vascular remodelling.

The miRNAs target a vast array of genes at varying efficiencies to fine‐tune the proteome and thereby regulate multiple processes concurrently (reviewed in 44). As well as targeting IGF‐1 and its receptor, miR‐424 has been shown to potentiate TGF‐β signalling via targeting of SMAD7 (a negative regulator of canonical TGF‐β signalling) in lung 45, mammary 46 and oesophageal epithelial cells 47. In this case, reduced miR‐424 might be expected to reduce TGF‐β‐induced hypertrophy 48, an effect opposite to that produced by regulating IGF‐1. However, in rat cardiac cells it has been shown that miR‐15b (a miRNA that shares the same seed sequence as miR‐322) targets components required for TGF‐β family signalling such as SMAD2/3 as well as SMAD7, suggesting that it may regulate the balance of TGF‐β and BMP signalling. Consequently, the effects of miR‐424 on TGF‐β/BMP signalling are likely to be cell type‐ and context‐specific. However, the specific capacity of miR‐322 to regulate these components is not known 34. Other validated targets for miR‐424 include cdc25, a component required to move through the cell cycle and apoptosis inhibitor 49, 50, and bcl‐2, an anti‐apoptotic protein upregulated in several cancers 51, 52. Recently, miR‐424‐5p was also shown to reduce rRNA production by targeting components of the RNA polymerase I pre‐initiation complex 53. Taken together, these data suggest miR‐424 is a promoter of cell quiescence and death, the loss of which can result in an increased proliferative capacity which can lead to an oncogenic phenotype, or in the case of the nondividing myocardium, hypertrophy.

## Conclusion

Our data suggest that reduced miR‐322‐5p contributes to the cardiac hypertrophy that occurs in PAH at least in part by increasing the expression of IGF‐1. This increase in ligand, in combination with the increase in receptor (IGF‐1R) that others have identified, increases the activity of the IGF‐1/Akt axis leading to increased protein synthesis and myocyte hypertrophy. These data suggest that miR‐322‐5p expression in normal circumstances acts to limit protein synthesis as part of the normal homoeostatic mechanisms. Whether this restriction on protein synthesis in normal physiology is removed only in response to pathological stimuli or whether the same mechanisms contribute to normal physiological hypertrophy in response to exercise remains to be determined.

## Author contributions

MC performed laboratory experiments and cowrote the first draft of the manuscript. PK and SJW conceived the research plan. PK cowrote the first draft of the manuscript. BEG, AC and NWM performed *in vivo* model and extracted tissues. All authors contributed critically to manuscript revisions.
